# High performance hot-deformed Nd-Fe-B magnets (Review)

**DOI:** 10.1080/14686996.2020.1868049

**Published:** 2021-01-28

**Authors:** Keiko Hioki

**Affiliations:** Corporate Research & Development Center, Daido Steel Co., Ltd., Nagoya, Japan

**Keywords:** Hot-deformed Nd-Fe-B magnet, heavy-rear-earth-element free, coercivity, microstructure, 106 Metallic materials

## Abstract

Hot-deformed anisotropic Nd-Fe-B magnets may potentially attain high coercivity due to their fine and highly orientated crystal grain microstructure as a result of the unique production process that creates these magnets. However, despite their fine grain size of 100–500 nm, coercivity was only around 25% of the full potential of the anisotropy field. This grain size was close to the critical diameter of the single domain grain size of the Nd-Fe-B magnet. This study investigated the effects of chemical composition and deformation conditions on the magnetic properties of Nd-Fe-B magnets, observing their microstructure to obtain guidance on the ideal microstructure. We also improved the hot-deformation technique in parallel to optimize microstructure by controlling the compositions and hot-deformation conditions based on the results of basic studies. Lastly, we fabricated heavy rare-earth-free magnets with a coercivity exceeding 1600 kA/m (20 kOe), which is 20% higher than that of conventional magnets.

## Graphical abstract

**Figure:**
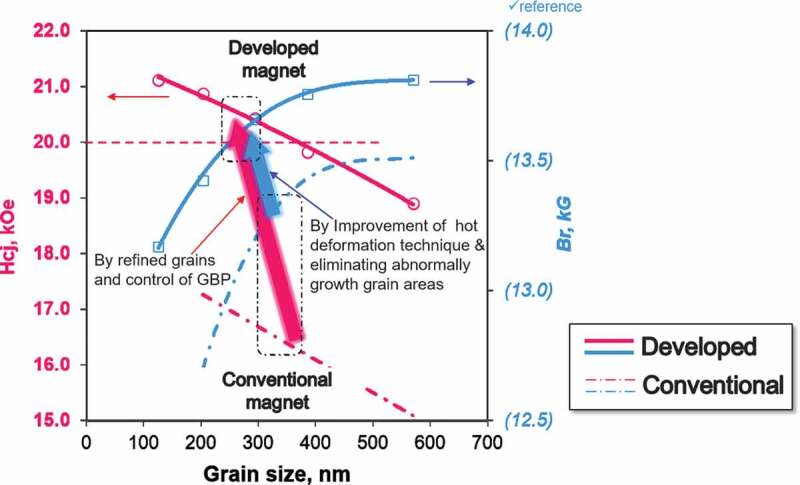

## 1. Introduction

Nd-Fe-B magnets with the highest maximum energy product (*BH*)_max_ of all permanent magnet materials were independently invented by Sagawa et al. [1] and Croat et al. [2] in 1982. Sintered Nd-Fe-B magnets by Sagawa [1] were the mainstream high-performance magnets because of their high magnetic properties and productivity. The rapid-quenched Nd-Fe-B powder by Croat et al. [2] was used as a raw material for bonded Nd-Fe-B magnets and hot-deformed Nd-Fe-B magnets [3]. Although sintered and hot-deformed Nd-Fe-B magnets have very similar magnetic properties, they differ in terms of their microstructures; hot-deformed Nd-Fe-B magnets have a fine microstructure (diameter of 200–500 nm), this is one order of magnitude finer than the sintered magnets.

Nd-Fe-B magnets have been widely used in clean energy applications such as hybrid electric vehicles (HEVs), consumer and other electronic devices, and wind turbines, because of their high magnetic performance. In particular, there has been an increasing demand for Nd-Fe-B magnets with high remanence and high heat resistance for HEV traction motors. Generally, these applications require magnets with high remanence, *B*_r_, and large coercivity, *H*_cj_. The addition of heavy rare-earth elements (HREEs), such as Dy or Tb, is the most common way to improve coercivity, as it increases the anisotropy field, *H*_a_ [4]. However, this addition reduces remanence and confronts resourcing issues as HREEs are scarce and produced from a limited area. Considering the ever-increasing demand for clean energy vehicles, the most important objective is to reduce HREE consumption in Nd-Fe-B magnets without leading to a significant reduction in coercivity.

To avoid this resource scarcity issue, there are two ways to improve coercivity with reduced or no HREEs; (1) utilizing the grain boundary diffusion (GBD) process [5,6], and (2) controlling the microstructure. The GBD process has been applied for sintered Nd-Fe-B magnets to effectively reduce HREE usage by more than 50%. Controlling the microstructure in terms of reducing grain size [7] and promoting grain isolation magnetically [8–10] have been generally known to increase coercivity. Thus, hot-deformed Nd-Fe-B magnets are considered a promising candidate to achieve completely HREE-free Nd-Fe-B magnets with high performance. The downside is that the coercivity of the hot-deformed magnet is not as high as expected given its fine grain size [11].

To address this, this study carried out a detailed investigation of the relationship between microstructures and the magnetic properties of hot-deformed magnets using systematic sample preparation and microstructure analysis. These results were used to optimize the chemical composition and hot-deformation conditions. This resulted in the production of HREE-free hot-deformed magnets with a coercivity exceeding 1600 kA/m; this is 20% higher than conventional magnets.

For readers’ better understandings, the author will show the basic information about the hot-formed magnet before describing the recent development studies in detail in section 4; production process will be shown in section 2 and feature of the magnet will be shown in section 3.

## 2. Production process of hot-deformed magnets

### 2.1. Production process

**Figure 1.**
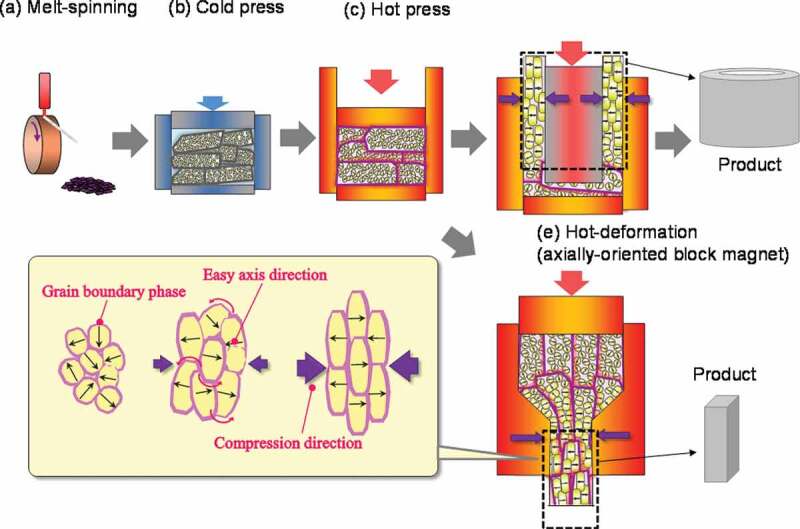
Production process of hot-deformed Nd-Fe-B magnet

Figure 1 presents a schematic of the manufacturing process for a radially oriented ring magnet (backward extrusion) and an axially oriented plate magnet (forward extrusion). The schematics in Figure 1(d, e) show the lateral profiles of both magnets, respectively. The magnet was fabricated by first preparing the starting material using a single-roller rapid quenching machine, followed by pulverization into a flake powder with a diameter of approximately 150 μm. This powder consists of numerous Nd_2_Fe_14_B nanocrystalline grains that were approximately 10–30 nm, with random orientation. The chemical composition of raw powder was slightly richer in rare-earth elements (REE) compared to the composition of the R_2_Fe_14_B crystal magnetic phase, in order to supply the liquid phase during hot deformation and grain boundary phase for final production. Then, the powder was cold-pressed at room temperature followed by hot-pressing at approximately 800°C to obtain a fully dense body. A hot-pressed body is an isotropic magnet with grains that are approximately 20–50 nm. Finally, to obtain anisotropic magnets, a hot-pressed body was hot-deformed at approximately 800°C; this is the point at which thermo-mechanical alignment of the crystal grains occurs. With the progression of the hot-deformation process, the grain size and its aspect ratio become large (diameter of 200–500 nm, thickness of 20–50 nm). The grains begin to align gradually, at the same time and the direction of the *c*-axis (easy axis) is perpendicular to the compression direction. The hot-pressing and hot-deformation temperatures were kept higher than the melting point of the grain boundary phase to ensure good formability whilst pressing. However, one of the reasons for abnormal grain growth was excess heat input during hot-pressing and hot-deformation [12,13]. Thus, it is important to fabricate this magnet with appropriate conditions and mold structures.

In Figure 1(d), the compression stress direction was radial during upward metal flow, resulting in a radially oriented ring magnet. This method enabled the uniform orientation of grains in the radial direction; this is advantageous for the production of small and large diameter and long ring magnets. It is also possible to fabricate the axially oriented block magnet by altering the mold structure and hot-deforming method described in Figure 1(e). Both types of hot-deformed magnets have already been mass produced.

### 2.2. Crystal orientation mechanism

Hot-deformation is usually completed in several tens of seconds, which means that significant microstructural change is completed within a short period of time. Specific mechanisms have been proposed to explain this phenomenon, although a consensus is still lacking on whether these mechanisms are indeed the cause of this phenomenon. In this section, we introduce our research group’s results and propose possible mechanisms from our results [14] and previous studies.

**Figure 2.**
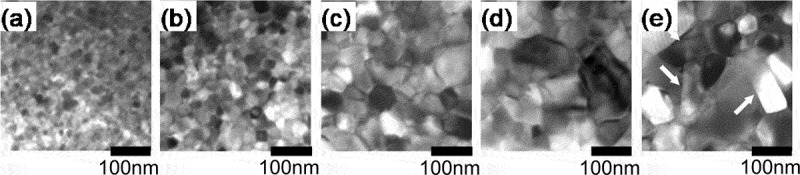
TEM imagery of rapid quenched ribbons annealed at 750°C for (a) 0 min (as quenched); (b) 1 min; (c) 3 min; (d) 5 min; and (e) 10 min (platelet grains are pointed by arrows) [14]

**Figure 3.**
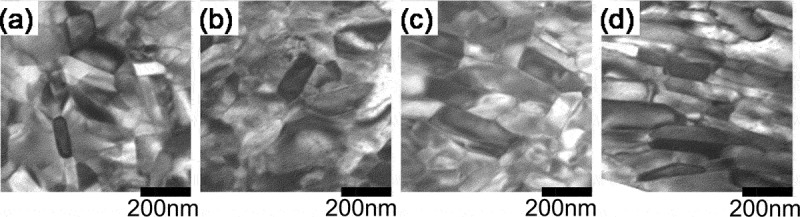
TEM imagery for die-upset magnets (perpendicular to compression force direction) with compression ratios, R, of (a) 0%; (b) 20%; (c) 40%; and (d) 60% [14]

Figure 2(a–e) shows the transmission electron microscopy (TEM) (Hitachi Ltd., Japan) images of raw powders before and after heat treatment [14]. Figure 3(a–d) presents the TEM images of die-upset samples that were perpendicular to the compression direction [14]. Here, the compression ratio, *R*, is defined by the equation below, where *h*_0_ and *h* are the height before and after die-upsetting:

(1) $$R=\;(h_{0}h)/h_{0}\times \;100\;\left(\%\right)$$

**Figure 4.**
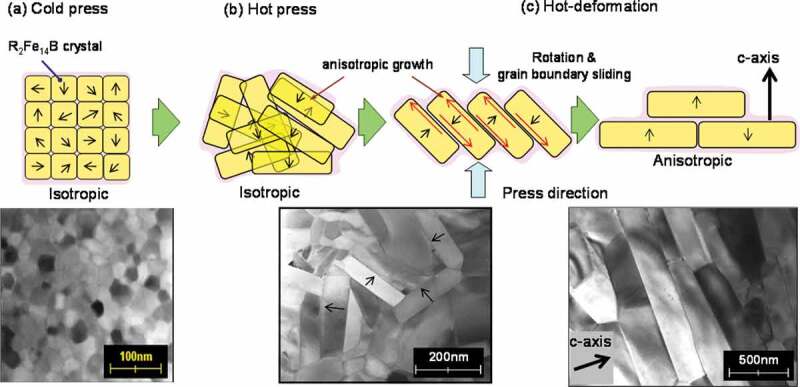
Schematic models of the change in microstructure for (a) cold-pressed body; (b) hot-pressed body; and (c) hot-deformed magnet

The schematic models of possible mechanisms our research group supports are described in Figure 4.

Figure 2 shows that the grains grow anisotropically with an increase in heat treatment time. The grain growth direction corresponds to the *a*-axis of the R_2_Fe_14_B crystal (tetragonal) [15]. Based on this result, the anisotropic growth direction of the R_2_Fe_14_B crystal was in the *a*-axis, as reported in previous studies [16]. In Figure 3(a–d), the grains align gradually in the same direction as the compression direction, as *R* became larger. The grain size and its aspect ratio simultaneously became larger, whilst no dislocations or slip lines were observed in normal area [13].

Therefore, we inferred possible mechanism as follows. First, the grain boundary phase melts and anisotropic grain growth occurs during hot pressing. Then, the anisotropic grain growth and crystal rotation with the grain boundary sliding of the c-plane (broad surface of platelet-shaped grain) are promoted by a stress under hot deforming [17]. Here, the liquid grain boundary phase acts as a lubricant and a path for the transfer of atoms which promotes the anisotropic grain growth under a compression stress. Finally, the *c*-axis of platelet grains align parallel to the press direction. It is conceivable the anisotropic grain growth during hot deformation is mainly caused by the atom transfer through the grain boundary phase because chemical composition of grain boundary phase changes during the microstructure evolution [18]. However, we do not understand completely the physical basis of this anisotropic grain growth under a compression stress at this time, though ‘grain boundary migration’ [12,13,17] and ‘interface-controlled solution-precipitation creep’ [19,20] have been proposed.

Another possible mechanism based on the elastic anisotropy of the R_2_Fe_14_B crystal was also proposed. In this mechanism, Young’s modulus of the *c*-axis is lower than the *a*-axis [21]; as such, grains grow easily along the *a*-axis under compression [22,23] and subsequently, such grains grow at the expense of other misaligned grains. However, as we recognize that the grains align gradually, we inferred that this mechanism was not dominant.

Based on the previous studies, it is apparent that one specific mechanism cannot completely account for the entire orientation phenomenon, although these mechanisms (and potentially an unknown new mechanism) are likely to be intricately related.

## 3. Features of hot-deformed magnets

### 3.1. Microstructure and magnetic property

**Figure 5.**
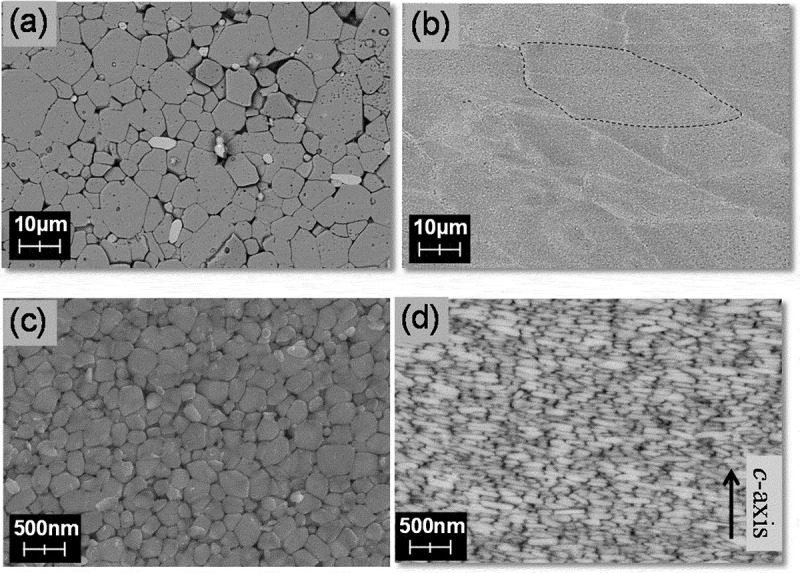
SEM images for (a) typical sintered magnet; and (b)–(d) hot-deformed magnet. Observed from the plane (b) and (c) parallel; and (d) perpendicular to the *c*-axis

Microstructure observations were conducted to understand the features of hot-deformed Nd-Fe-B magnets. Figure 5(a–d) show microstructures of sintered and hot-deformed Nd-Fe-B magnets observed by SEM (JEOL Ltd., Japan), respectively. The composition of samples is shown in Table 1. Figure 5(a,b) were observed at the same magnification to compare microstructures. The dashed line in Figure 5(b) indicates the boundary of the original raw powder and Figure 5(c, d) shows that the microstructures were parallel and perpendicular to the *c*-axis, respectively. As shown in Figure 5(c, d), there were numberless platelet-shaped grains stacked in the *c*-axis direction; this is one order of magnitude finer than the sintered magnet. The grain size was similar to the critical diameter of single-domain Nd_2_Fe_14_B grains (0.3 μm) [24].

**Table 1.** Chemical compositions of sintered and hot-deformed magnets in Figures 5–8 and 14

		Chemical composition (wt.%)								
		TRE	Nd	Pr	Dy	Fe	Co	B	Ga	Al,Cu
Figure 5(a) S	S	31.0	25.9	<0.1	5.1	bal.	2.4	0.9	0.0	0.5
Figure 5(b-d), 7, 8(b, c), 14(a-c)	HD	29.8	29.8	<0.1	0.0	bal.	3.4	0.9	0.6	<0.1
Figure 6(a, b)	S	30.5	26.2	0.2	4.1	bal.	2.2	1	0.0	0.5
Figure 6(b)	S	30.7	22.1	0.1	8.5	bal.	2.2	1	0.0	0.4
Figure 6 (a, b)	HD	30.4	25.9	0.1	4.2	bal.	3.5	0.9	0.5	<0.1
Figure 7, 8(a)	S	29.3	22.4	6.3	0.6	Bal.	2.0	0.9	0.0	0.5

bal. stands for balance. S and HD correspond to sintered and hot-deformed magnets, respectively.

**Figure 6.**
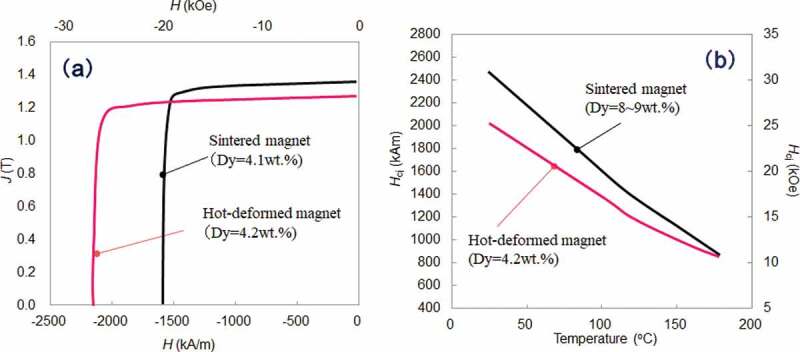
(a) Demagnetization curves; and (b) temperature dependence of coercivities for sintered and hot-deformed Nd-Fe-B magnets. Sintered magnets were not GBD processed samples. The Dy amount of each magnet is described in the figures

**Figure 7.**
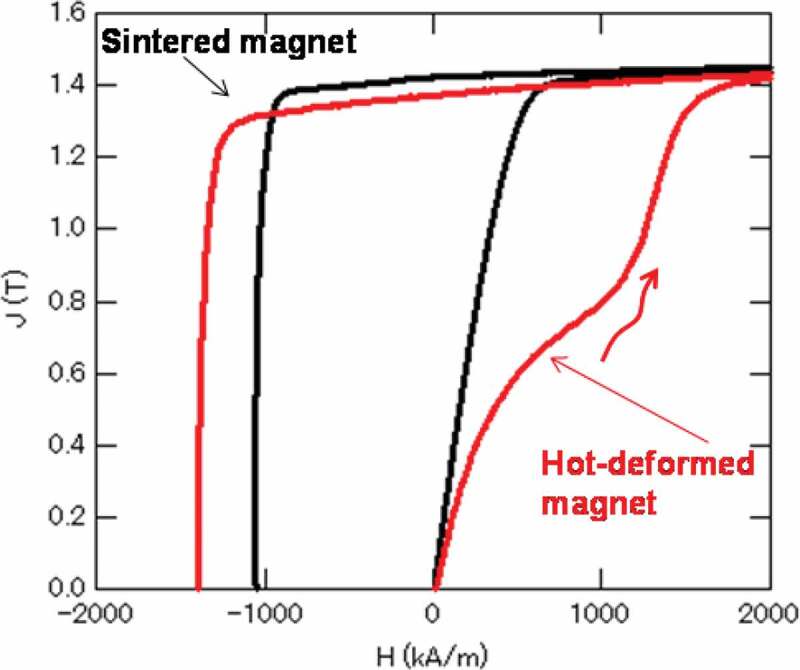
Initial magnetization and demagnetization curves of typical sintered and hot-deformed Nd-Fe-B magnets

**Figure 8.**
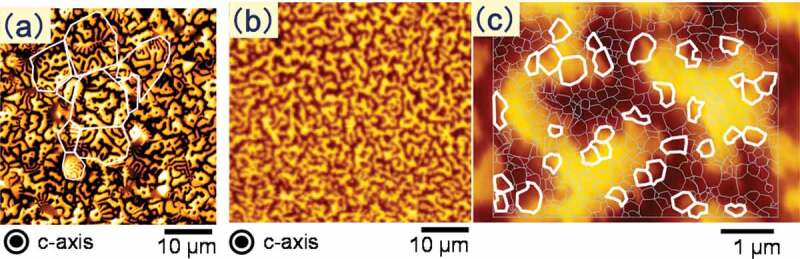
Magnetic domain pattern images of typical (a) sintered; and (b)–(c) hot-deformed Nd-Fe-B magnets observed by magnetic force microscopy (MFM). The contours of grains obtained from LV-SEM observations were overlaid in (a) and (c)

Figure 6(a, b) shows the demagnetization curves and temperature dependence of the coercivities for sintered and hot-deformed Nd-Fe-B magnets measured by BH tracer and Pulsed high field magnetometer (Toei Industry Co., Ltd., Japan), respectively. The composition of samples is shown in Table 1. Despite having very similar Dy concentrations, the coercivity of the hot-deformed magnet was higher than the sintered magnet, due to the difference in the microstructures. The remanence of the hot-deformed magnet was lower than the sintered magnet because the grain alignment and the volume of main phase of the latter were higher than that of the hot-deformed magnet. Here, the difference of volume fraction between these samples can be interpreted as the difference of B wt.%. In Figure 6(b), temperature dependence of the coercivities for the same samples as Figure 6(a) is shown. The difference of coercivity between the sintered and hot-deformed magnets is a bit larger at 180°C than that at room temperature. For reference, the sintered magnet with Dy = 8.5 wt.% is shown in Figure 6(b) together. It is clear that it demonstrated higher coercivity at room temperature than the hot-deformed magnet with Dy = 4.2 wt.%, while they both had the same coercivity at 180°C. These results show that the hot-deformed magnet exhibits higher coercivity and heat resistance than its sintered counterpart.

### 3.2. Initial magnetization curve

Figure 7 shows the initial magnetization and demagnetization curves of typical sintered and hot-deformed Nd-Fe-B magnets, respectively. The composition of samples is shown in Table 1. Figure 7 shows that the sintered magnet was easily magnetized under a low magnetic field. In contrast, the initial magnetization curve of the hot-deformed magnet exhibited a two-step process with two inflection points, indicating that this magnet has two different magnetic phases. The initial magnetization curve of the second step is considered to indicate the magnetization process of the pinning-type grains. It is inferred that such a pinning-type grain is a single-domain grain, while a recent study reported that some multi-domain grains do not reverse until the applied magnetization field is close to its coercivity [25]. The initial first-step magnetization curve was magnetized at a lower applied magnetic field, indicating that the magnetic domain wall moves easily in the grains. In addition, single-domain grains whose grain boundary phase is poor or high magnetization would also behave similar to multi-domain grains as a group. The relationship between the microstructure and initial magnetization curve is shown in 4–1.

### 3.3. Magnetic domain pattern

Figure 8(a–c) show the magnetic domain pattern images of typical sintered and hot-deformed Nd-Fe-B magnets, respectively, as observed by magnetic force microscopy (MFM) (Hitachi High-Tech Science Corp., Japan). The composition of samples is shown in Table 1. In this instance, the magnets were in thermally demagnetized states and the plane of observation was the c-plane. The images in Figure 8(a, b) were observed at the same magnification, and Figure 8(c) shows the MFM images of the insides of the powder in Figure 8(b). Contours of grains obtained from the low-voltage scanning electron microscopy (LV-SEM) observation, drawn with white lines, are overlaid in Figure 8(a, b). The bright and dark shaded areas represent two magnetic polarizations, N and S, respectively.

In Figure 8(b, c), the magnetic domain pattern of the hot-deformed magnet exhibits a maze pattern that was identical to that of the sintered magnet in Figure 8(a). However, it was found that the groups of single-domain grains form a magnetic domain pattern in the hot-deformed magnet [26,27]. Relatively large grains at the edges of the maze pattern are multi-domain grains and are outlined in white. The edges of the maze pattern were not smooth, reflecting the shape of single domain grains. It is inferred that such a magnetically mixed structure which consists of single-domain grains and multi-domain grains has higher coercivity and heat resistance. Details are shown in 4–1.

## 4. Recent developments in HREE-free/HREE-reduced hot-deformed magnets

### 4.1. The effect of microstructure and composition on magnetic properties

The effects of chemical composition and microstructure (grain size, composition of grain boundary phase, grain boundary size), on the magnetic properties of hot-deformed Nd-Fe-B magnets were systematically evaluated [28]. Table 2 shows the chemical composition and process conditions that had been utilized. Samples A–C were prepared to investigate the effects of grain size and grain boundary phase conditions, and samples D and E were prepared to determine the effect of Dy addition; the detailed sample preparation process is provided in ref [28].

**Table 2.** Chemical compositions and hot-deformation temperatures of samples[26]

	Chemical composition (at.%)						Hot-deformation temperature (^o^C)
	Nd	Dy	Fe	Co	B	Ga	
A	12.8	0.0	bal.	3.85	5.65	0.46	750,775,800,825,850,875,900
B	13.5	0.0	bal.	3.82	5.64	0.57	750,775,800,825,850,875
C	14.2	0.0	bal.	3.81	5.66	0.71	725,750,775,800,825,850
D	12.4	1.0	bal.	3.85	5.59	0.57	775,800,825,850,875,900
E	11.5	2.0	bal.	3.91	5.56	0.58	750,775,800,825,850,875,900

bal. stands for balance.

**Figure 9.**
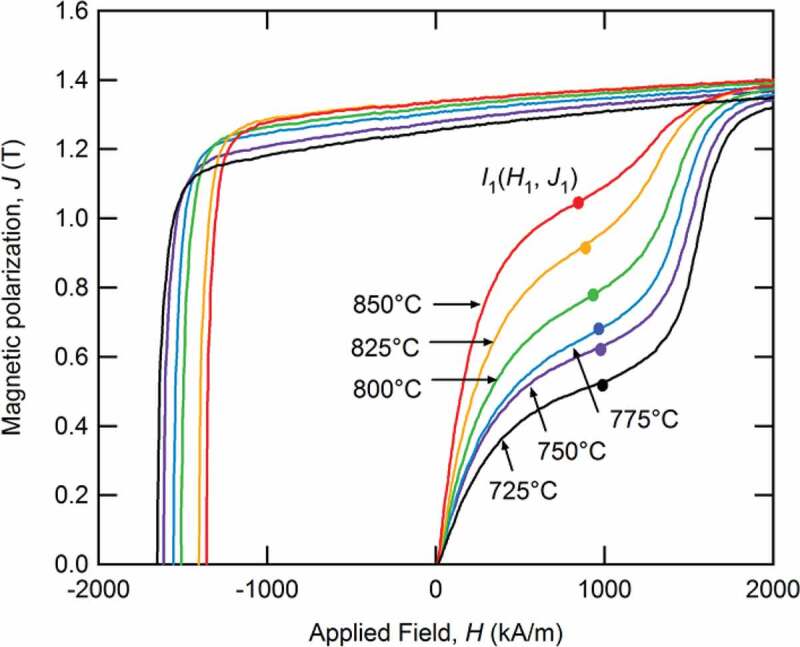
Initial magnetization and demagnetization curves for Sample C following deformation at 725°C, 750°C, 775°C, 800°C, 825°C, and 850°C [26]

Figure 9 shows the magnetization curves of sample C, which was hot-deformed at various temperatures. This figure shows that the coercivity becomes higher as the hot-deformation temperature decreases, and remanence increases with the hot-deformation temperature; other samples exhibited the same trend. In addition, the shape of the initial magnetization curves which reflect the magnetic structure as mentioned in 3–2 is also changed gradually. Hence, the single-domain grain ratio (SDGR) can be estimated as eq.(2),

(2) $$SDGR=1-J_{1}/J_{S}$$

*J*_1_ is the inflection point of initial magnetization curves and *J*_S_ is the saturation magnetization.

**Figure 10.**
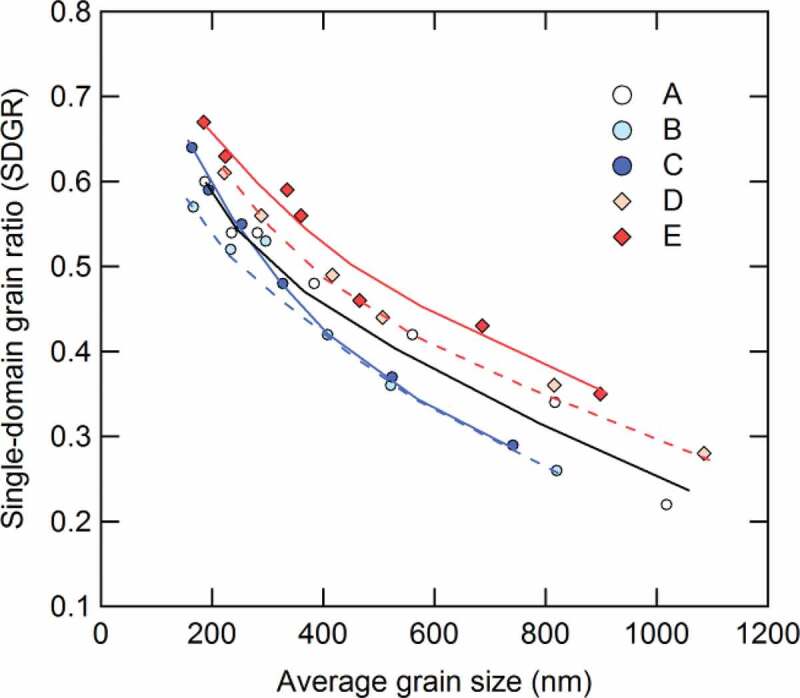
Dependence of the single-domain grain ratio (SDGR) on the average grain size for samples A–E [26]

Figure 10 shows the relationship between average grain size and SDGR in samples A–E. Here, the grain sizes are defined perpendicular to the thickness direction of the platelet-shaped Nd_2_Fe_14_B grains. The SDGRs of all samples A–E increase as the average grain size decreases, which indicates the increasing volume of single-domain structure corresponds to decreasing average grain size.

**Figure 11.**
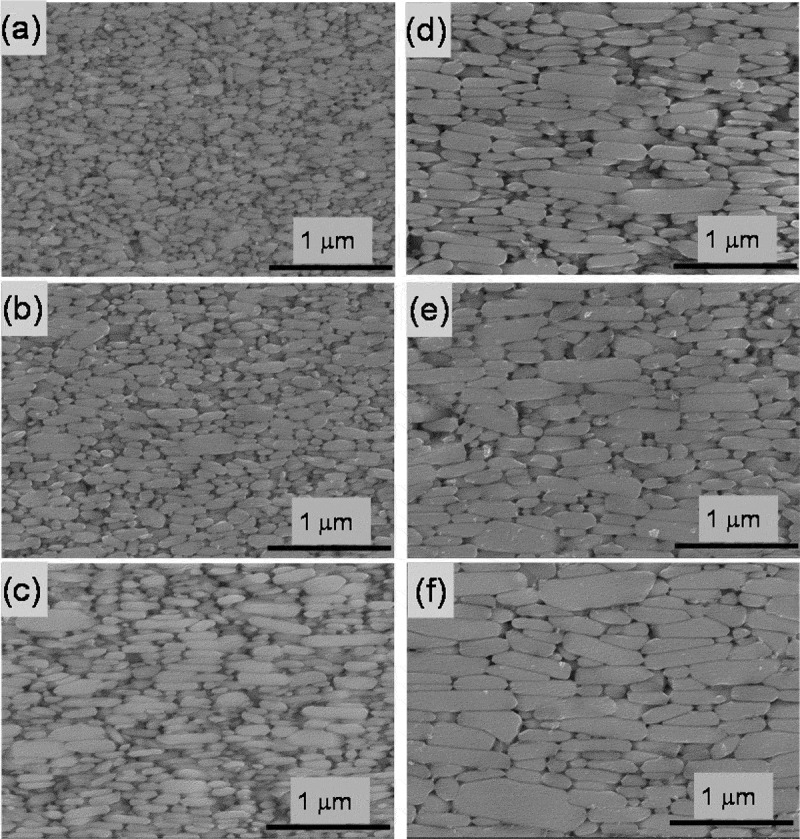
SEM photographs parallel to the c-axis for Samples C after deformation at (a) 725^o^C; (b) 750°C; (c) 775°C; (d) 800°C; (e) 825°C; and (f) 850°C [26]

Figure 11(a–f) presents the SEM imagery for sample C that had been hot-deformed from 750°C to 875°C. The observation planes are parallel to the *c*-axis. As seen in the figure, it is clear that the crystal grains grow as the hot-deformation temperature increases.

**Figure 12.**
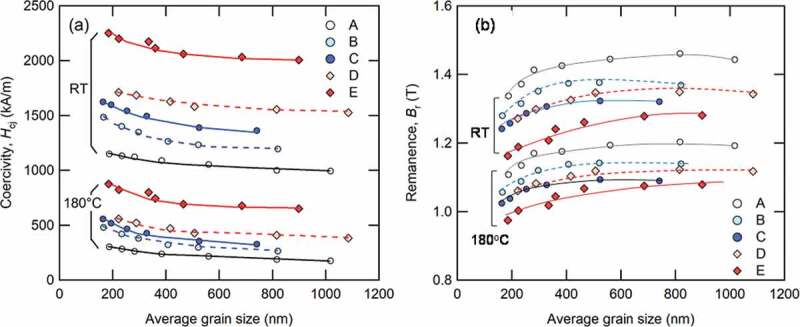
Dependence of (a) coercivity; and (b) remanence on average grain size for Samples A–E at room temperature and 180°C. Lines provide visual guides [26]

Figure 12(a, b) shows how average grain size is dependent on coercivity and remanence at room temperature and 180°C. As shown in Figure 12(a), the coercivities of all samples increase with decreasing average grain size. This figure also shows that adding Dy and increasing the amount of TRE improves coercivity. It should be emphasized that the coercivities of Sample C (Dy-free), whose average grain size was less than 400 nm, were comparable with those of Sample D (Dy = 1.0 at.%). In Figure 12(b), the remanence decreases with decreasing grain size; this is because grain alignment requires a certain level of anisotropic grain growth, as discussed in Section 2–2. Additionally, the hot-deformation temperature was too high, and remanence also decreased in this instance because the abnormal coarse grains obstruct grain alignment due to excess heat input.

**Figure 13.**
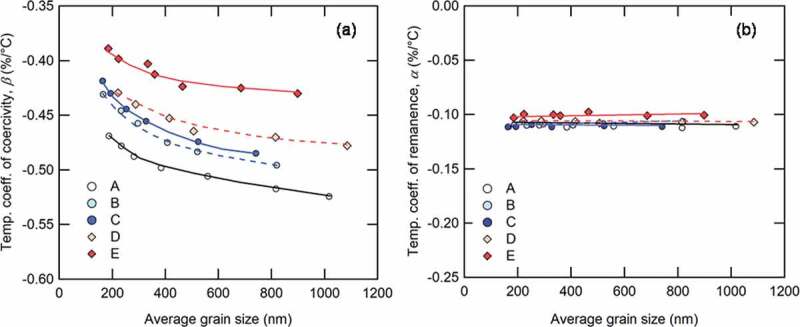
Dependence of (a) temperature coefficient of coercivity; and (b) temperature coefficient of remanence on average grain size for Samples A–E. Lines provide visual guides [26]

Figure 13(a, b) shows the dependence of temperature coefficient of coercivity and remanence, *β* and *α*, between room temperature and 180°C for all samples, where α and *β* are given by:

(3) $$\beta =\frac{{\;}_{i}H_{c}\left(23^{\circ }C\right)-{\;}_{i}H_{c}\left(180^{\circ }C\right)}{\left(23^{\circ }C-180^{\circ }C\right)}\times \frac{100}{{\;}_{i}H_{c}\left(23^{\circ }C\right)}$$

(4) $$\alpha =\frac{B_{r}\left(23^{\circ }C\right)-B_{r}\left(180^{\circ }C\right)}{\left(23^{\circ }C-180^{\circ }C\right)}\times \frac{100}{B_{r}\left(23^{\circ }C\right)}$$

From Figure 13(b), it is apparent that the *α* values of all samples were around −0.1%/°C; they are unaffected by either grain size or the TRE. On the other hand, the *β* values of all samples improved with the addition of Dy, the increased TRE content, and the grain size reduction. This result shows that it is possible to obtain high heat resistance without HREEs by reducing grain size. Besides, it was found that the increasing volume of single-domain structure affects not only coercivity but also improvement of the *β* values from Figures 10, 12 and 13.

Liu et al. [29] made microstructural observations of Dy-free hot-deformed magnets of Nd = 12.7, 13.0, and 14.0 at.% to clarify the relationship between the grain boundary phase conditions and magnetic properties. The Nd amounts of these three samples were close to Samples A–C in Table 1. Their coercivities were *H*_cj_ = 720 (9.0 kOe), 1030 (12.9 kOe), and 1430 kA/m (17.9 kOe), respectively. The coercivity increased with the Nd amount, as observed for Samples A–C. They observed the thickness and chemical composition of the grain boundary phase using TEM, LV-SEM, and three-dimensional atomic probe (3DAP). The observations showed that the thickness of the grain boundary phases increased with the Nd content. In this study, the average Nd content in the grain boundary phase increased from 22.7.% to 46.0 at.% with an increase in thickness. These results suggest that the increase of Nd at the grain boundary phase enhances the magnetic isolation of the grains.

Another microstructural analysis of the effect of grain size was also reported. Sepheri-Amin et al. [30] observed the microstructure of Dy-free hot-deformed magnets of Nd = 13.0 at.% fabricated at various temperatures. The chemical compositions of their samples were almost the same as those of Sample B in Table 2. As the hot-deformation temperature increased, the coercivity decreased similar to Figure 12(b). As a result of these microstructure observations, it was clarified that samples hot-deformed at a low temperature had a finer crystal structure, a smaller crystal grain size distribution, and higher Nd content in the intergranular boundary phase. On the other hand, the sample that had hot-deformed at a high temperature had not only coarse grains but also large triple junctions; as a result, the intergranular grain boundary phase became thin.

These studies found that there were improvements to the heat resistance of hot-deformed magnets through the control of the microstructure without the addition of HREEs. Some simulations also support these experimental results [29–32].

### 4.2. Improving uniformity of microstructure

**Figure 14.**
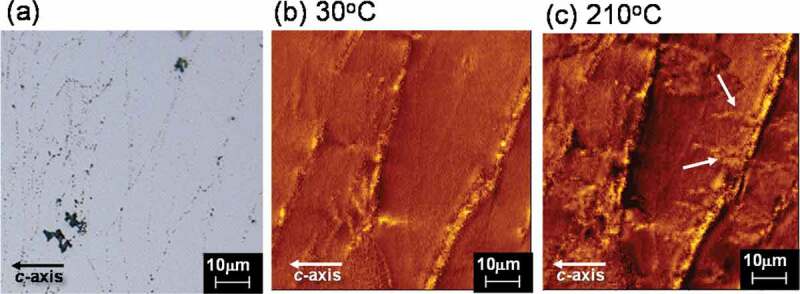
(a) Microstructure; and (b)–(c) magnetic domain pattern during the thermal demagnetization process observed by MFM at 30°C and 210°C

**Figure 15.**
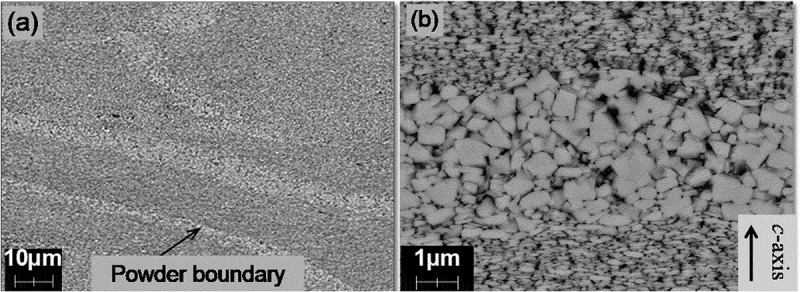
Microstructure at a raw powder boundary equivalent to where magnetization reversal occurs in Fig. 14 (c)

A change in the magnetic domain pattern during the thermal demagnetization process was observed, to identify the starting point where magnetization reversal occurs. The composition of the sample is shown in Table 1. In-situ MFM imagery of the samples was conducted during thermal demagnetization at 30°C and 210°C, as shown in Figure 14. The sample was a typical hot-deformed magnet, and the observation plane was parallel to the *c*-axis. The sample was first fully magnetized, such that almost all grains were magnetized in the same direction at 30°C (Figure 14(b)). With an increase in temperature, the reverse magnetization areas (shown by the white arrows) from the powder boundary were observed (Figure 14(c)). Figure 15 shows the microstructure at a raw powder boundary, equivalent to where magnetization reversal occurs in Figure 14(c). These results showed that magnetization reversal occurs from the area corresponding to relatively coarse and misaligned grains.

**Figure 16.**
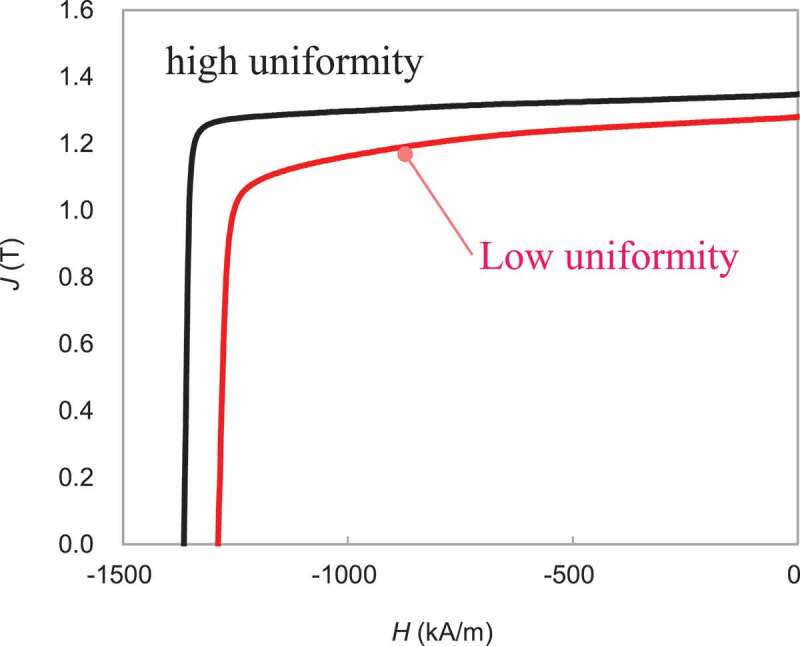
Demagnetization curves for hot-deformed magnets before and after the reduction of coarse and misaligned grains at powder boundaries

Figure 16 presents the demagnetization curves for samples before and after the reduction of coarse and misaligned grains at powder boundaries by controlling hot-deformation conditions. As a result of improvements to the uniformity of entire microstructures, coercivity and remanence had been enhanced, and the squareness of the demagnetization curve had also improved. By refining crystal grain size and increasing the amount of grain boundary phase described in Section 4–1, coercivity could be improved with the decrease of remanence to some extent. However, this decrease in remanence decrease may be avoided by improving the uniformity of the microstructure.

### 4.3. The latest property map

**Figure 17.**
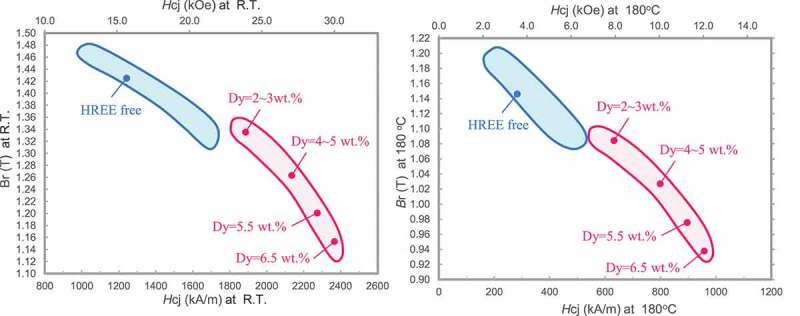
Magnetic property maps for hot-deformed magnets at (a) room temperature; and (b)180°C

Figure 17(a, b) shows the magnetic property map of Dy-free and Dy-saving hot-deformed Nd-Fe-B magnets measured at room temperature and 180°C, respectively. Improvements to the microstructure, as discussed in Sections 4–1 and 4–2, lead to enhanced magnetic properties in this magnet. In particular, the coercivity of the HREEs-free hot-deformed Nd-Fe-B magnets exceeded 1600 kA/m, which have been used for traction motors in HEVs [33].

## 5. Commercial products

**Figure 18.**
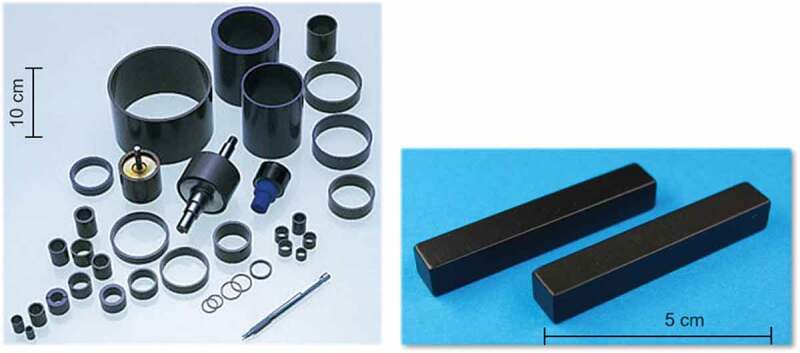
Products of the radially oriented ring and axially-oriented plate hot-deformed Nd-Fe-B magnets

Figure 18 presents examples of products from radially oriented ring and axially oriented plate hot-deformed Nd-Fe-B magnets. The features of these magnets include:

1. A ring magnet with a wide range of diameters (minimum outer diameter = 5 mm) and lengths (maximum 80 mm) may be produced;
2. The magnetic flux is uniform along the height direction even if the ring magnet has a great height. This is because it does not require grain alignment under a magnetic field;
3. Various magnetization patterns and precise magnetization waveform control were possible by controlling mold structure;
4. Relatively good corrosion resistance; and [34]
5. Near net-shape manufacturing was possible.

## 6. Conclusion and future development

In order to improve the coercivity of hot-deformed Nd-Fe-B magnets without HREEs addition, we investigated the effects of chemical composition and hot deformation conditions on the magnetic properties of the magnets, observing their microstructures to obtain guidance on the ideal microstructure. Based on these results, we optimized compositions and hot-deformation conditions to enhance the coercivity by refining grains and promoting grain isolation magnetically and to improve the remanence by eliminating abnormally growth grain areas. Consequently, we successfully developed high performance hot-deformed magnets that had greater than 1600 kA/m (20 kOe) of coercivity without any addition of HREEs, which are used for HEV traction motors [33]. However, further developments are required to respond to the new requirements for next-generation application designs from customers. The examples of this include:

1. To improve the accuracy in controlling the microstructure, further investigation of the orientation mechanism as described in Section 2–2 is required. If the mechanism is completely clarified, it will be possible to construct the optimum profile of hot-deformation to control the microstructure more than ever;
2. To provide magnets for newly designed motors [35], hot-deformation techniques are developed to fabricate various magnet shapes with various grain orientations;
3. Application of grain boundary engineering techniques such as the GBD process to hot-deformed magnets for a remarkable increase in coercivity with small HREE addition [36–38];
4. Recently, it has been possible to experimentally observe the magnetization reversal of a few particles [39]. From such studies, to obtain the ideal microstructure to fully understand the potential of hot-deformed magnets;
5. To avoid resource problems, low-cost REEs such as Ce and La need to be used efficiently. It is clear that the magnetic properties become worse by substituting them with Nd. However, there are reports that the magnetic properties do not worse as much as the physical properties by optimizing composition and microstructure [40,41]. For hot-deformed magnets, it is possible to improve the formability of hot-deformed magnets as the melting point of the grain boundary phase decreases due to Ce; this has a positive effect on the alignment grains and productivity [42,43];
6. Increase the electrical resistivity of the magnet material. During motor operation, the temperature of the magnets increases due to the eddy current at the surface of the magnets. The higher electrical resistivity of magnets is effective for reducing eddy currents. For hot-deformed magnets, there are studies investigating into mixing high-electrical resistivity material with raw powder, followed by fabricating magnets [44,45]. However, it appears that there is difficulty in increasing the electrical resistivity by suppressing the deterioration of magnetic properties.
